# Galanin Regulates Myocardial Mitochondrial ROS Homeostasis and Hypertrophic Remodeling Through GalR2

**DOI:** 10.3389/fphar.2022.869179

**Published:** 2022-03-31

**Authors:** Frederic Boal, Mathieu Cinato, Andrei Timotin, Heike Münzberg, Emily Qualls-Creekmore, Solomiia Kramar, Halyna Loi, Jerome Roncalli, Sokhna Keita, Helene Tronchere, Oksana Kunduzova

**Affiliations:** ^1^ National Institute of Health and Medical Research (INSERM) U1297, Toulouse, France; ^2^ Paul Sabatier University, Toulouse, France; ^3^ Neurobiology of Nutrition and Metabolism Department, Pennington Biomedical Research Center, Louisiana State University System, Baton Rouge, CA, United States; ^4^ Department of Cardiology, Toulouse University Hospital, Toulouse, France

**Keywords:** galanin, galanin receptors, oxidative stress, hypertrophy, cardiac remodeling

## Abstract

The regulatory peptide galanin is broadly distributed in the central nervous systems and peripheral tissues where it modulates numerous physiological and pathological processes through binding to its three G-protein-coupled receptors, GalR1-3. However, the function and identity of the galaninergic system in the heart remain unclear. Therefore, we investigated the expression of the galanin receptors in cardiac cells and tissues and found that GalR2 is the dominant receptor subtype in adult mouse hearts, cardiomyocytes and H9C2 cardiomyoblasts. *In vivo*, genetic suppression of GalR2 promotes cardiac hypertrophy, fibrosis and mitochondrial oxidative stress in the heart. *In vitro*, GalR2 silencing by siRNA abolished the beneficial effects of galanin on cell hypertrophy and mitochondrial reactive oxygen species (ROS) production. These findings unravel new insights into the role of galaninergic system in the heart and suggest novel therapeutic strategies in heart disease.

## Introduction

Heart failure (HF) is a common denominator of acute and chronic heart diseases and one of the leading causes of morbidity and mortality worldwide (Kamran and Tang, 2021). Despite significant advances in treatment and prevention, patients with HF continue to face unacceptably high rates of hospitalization and death reflecting inadequacy of modern therapy strategies. The onset of HF is typically preceded by hypertrophy and mitochondrial defects, the major features of myocardial remodeling, which results in function decline (Sutton and Sharpe, 2000; Konstam et al., 2011; Schiffrin, 2012; Passino et al., 2015). Cardiac hypertrophy is characterized by an increase in cardiomyocyte size and thickening of ventricular walls. Initially, myocyte growth is an adaptive response to maintain cardiac function; however, in settings of sustained stress, hypertrophic remodeling become maladaptive and predispose to cardiovascular morbidity and mortality (Bernardo and McMullen, 2016). Cardiac hypertrophy is closely linked to oxidative stress and mitochondrial damage in HF phenotype (Heineke and Molkentin, 2006), (Rosca et al., 2013). However, the complex molecular and cellular mechanisms underlying abnormal hypertrophic remodeling and mitochondrial homeostasis remain largely obscure.

Galanin is a 29-amino or 30 amino acid peptide naturally present in the tissues and fluids of mammals (Crawley, 1995). Galanin is widely distributed in the central nervous system and peripheral tissues. The amino acid sequence of galanin is very conserved among species (almost 90%), indicating the importance of this peptide (Crawley, 1995). Galanin orchestrates numerous physiological functions, including nociception, sleep regulation, cognition, energy homeostasis, neuroendocrine activities and central cardiovascular control (Crawley, 1995; Díaz-Cabiale et al., 2005; Díaz-Cabiale et al., 2010). Galanin is linked to a number of diseases including epilepsy, depression and eating disorders (McDonald and Crawley, 1997; Lerner et al., 2010; Zhao et al., 2013; Millón et al., 2017). We have recently demonstrated that galanin exhibits cardioprotective properties against ischemia-reperfusion (I/R)-mediated cardiac injury in animals (Pisarenko et al., 2017; Martinelli et al., 2021). Administration of this peptide limited myocardial infarct size and improved metabolic features *in vivo* (Timotin et al., 2017). Galanin triggers cellular responses by activating three distinct G-protein-coupled receptors, galanin receptor 1 (GalR1), GalR2, and GalR3, which differ in their pharmacology, signaling, and distribution. Among these receptors, GalR1 and GalR3 mainly activate G_i/o_ types of G proteins, mediating inhibitory actions of galanin (Habert-Ortoli et al., 1994; Burgevin et al., 1995; Wang et al., 1997). In contrast, the GalR2 subtype can transmit either stimulatory effects of galanin, for example on neurotransmitter release, acting *via* G_q/11_ types of G proteins, or it can inhibit neurotransmission *via* G_i/o_ types (Fathi et al., 1998; Branchek et al., 2000; Shen et al., 2005). Galanin and its receptors have also been associated with regulation of neurogenesis, stroke-related damage, inflammation, diabetes and cancer (Berger et al., 2005; Lang and Kofler, 2011; Fang et al., 2016). However, the expression and functional activity of galanin receptors in the heart remains largely unexplored.

Here we report that GalR2 is the predominant receptor subtype in the heart that transduces the protective effects of galanin in cardiac cells. Genetic suppression of GalR2 promotes activation of hypertrophic remodeling and mitochondrial oxidative stress in mice.

## Materials and Methods

### Histology

Hematoxylin-eosin (H&E) and Masson’s Trichrome staining were performed on 10 μm heart cryosections at the Histology Facility according to standard methods. Wheat germ agglutinin (WGA) staining was performed on PFA-fixed cryosections after Triton X-100 permeabilization using Oregon Green-coupled WGA (ThermoFisher Scientific as described before (Cinato et al., 2021). Myocytes cross-sectional area was manually quantified in a blinded manner using ImageJ software on WGA-staining sections. The extent of cardiac structural changes was quantified using ImageJ software.

### Mutant Mice Deficient for GalR2

Mice aged 8–10 months deficient for the GalR2 gene (GalR2mut) were derived from the colony of Marina Picciotto (Yale University, New Haven, CT, United States), where they were backcrossed onto a C57Bl/6J background for at least 10 generations as described previously (Hobson et al., 2006; Einstein et al., 2013). Homozygotes mice were used in the study. Breeding and experimental procedures using GalR2 wild type and GalR2mut mice were approved by the Institutional Animal Care and Use Committee at the Pennington Biomedical Research Center.

### Cell Culture, Transfection and Treatments

The rat embryonic cardiomyoblastic cell line H9C2 (ATCC) was cultured as described (Tronchere et al., 2017) in DMEM medium supplemented with 10% FBS and 1% penicillin-streptomycin in a 37°C, 5% CO_2_ incubator. siRNA transfection was performed with Lipofectamine RNAiMAX (Life Technologies) according to manufacturer’s instructions. Briefly, cells seeded in 500 µl medium in 24-well plates were transfected using 45 pmol siRNA with 1 µl LipoRNAiMAX in 100 µL OptiMEM. Twenty-4 hours later, cells were pre-treated with 10 nM galanin for 30 min and then stimulated with 5 µM isoproterenol (ISO) for 4 h (to assess mitochondrial O_2_^−^ production) or for 48 h to address cell hypertrophic response.

### Molecular Biology

**FIGURE1:**
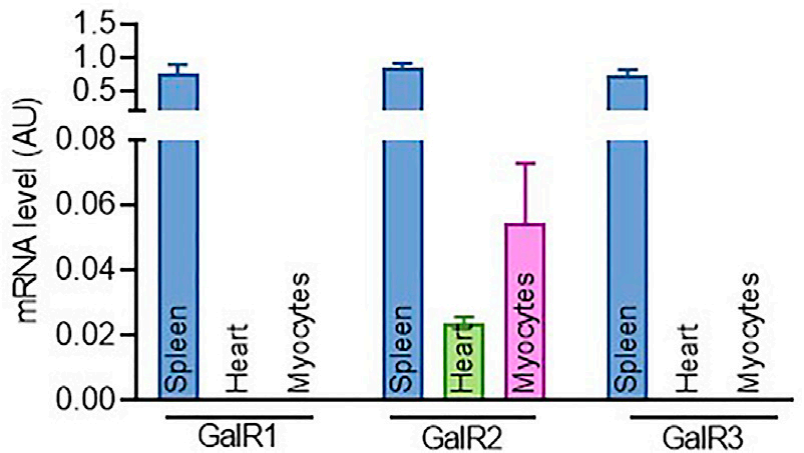
Expression profiling of GalR1, GalR2 and GalR3 in cardiac tissues and cells. The expression level of GalR1, GalR2 and GalR3 mRNA in mouse spleen, heart and primary cardiomyocytes. Data are presented as the mean ± SEM from at least three independent experiments.

**FIGURE 2:**
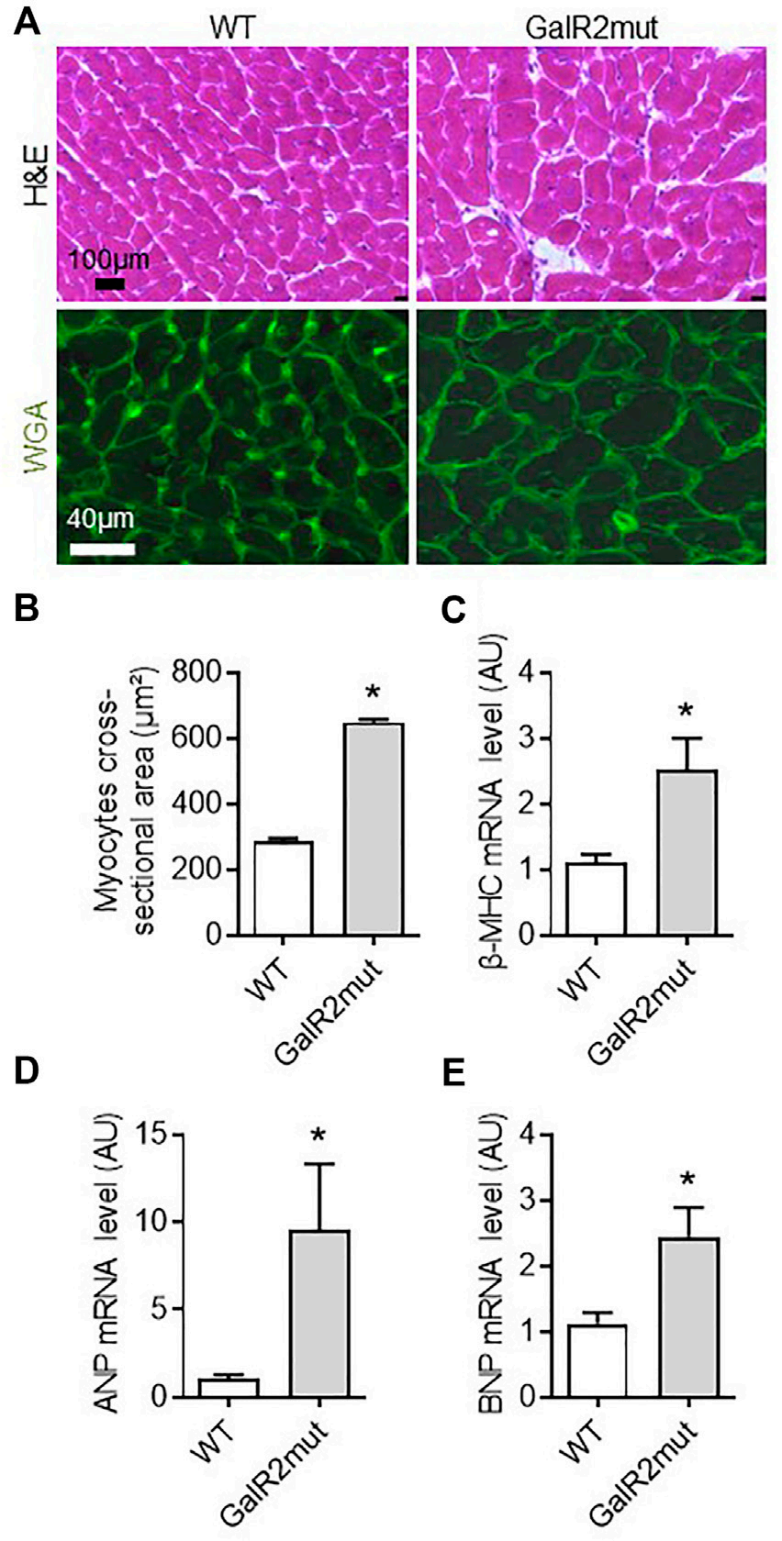
Cardiac hypertrophic phenotype in GalR2 deficient mice. Representative images of cardiac sections stained with H&E and WGA **(A)** and quantification of the cross-sectional area of cardiomyocytes from A in WT and GalR2mut mice **(B)**, *n* = 5–6 mice. Myocardial mRNA levels of the hypertrophic markers (β-MHC, ANP, and BNP) in WT and GalR2-Mut mice **(C–E)**, *n* = 7–9 mice. Data are presented as the mean ± SEM, *p < 0.05 vs. WT.

**FIGURE 3:**
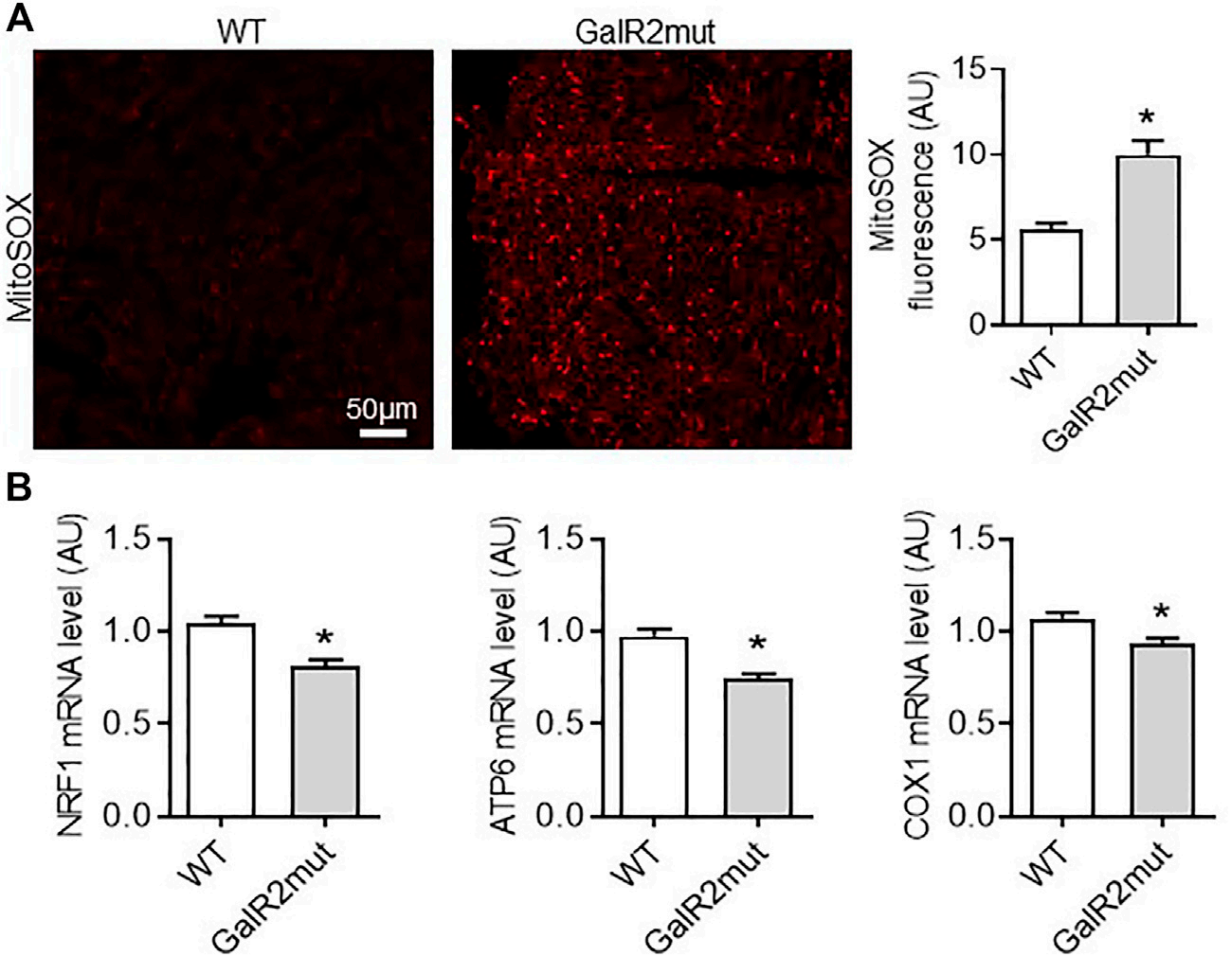
Cardiac mitochondrial ROS status in GalR2 deficient mice. **(A)** Representative images and quantification of cardiac sections stained with MitoSox Red from WT and GalR2mut mice, *n* = 4–6 mice. **(B)** Myocardial mRNA levels of NRF1, ATP6 and COX1 in WT and GalR2mut mice, *n* = 5–7. Data represents the mean ± SEM. *p < 0.05 vs. WT.

**FIGURE 4:**
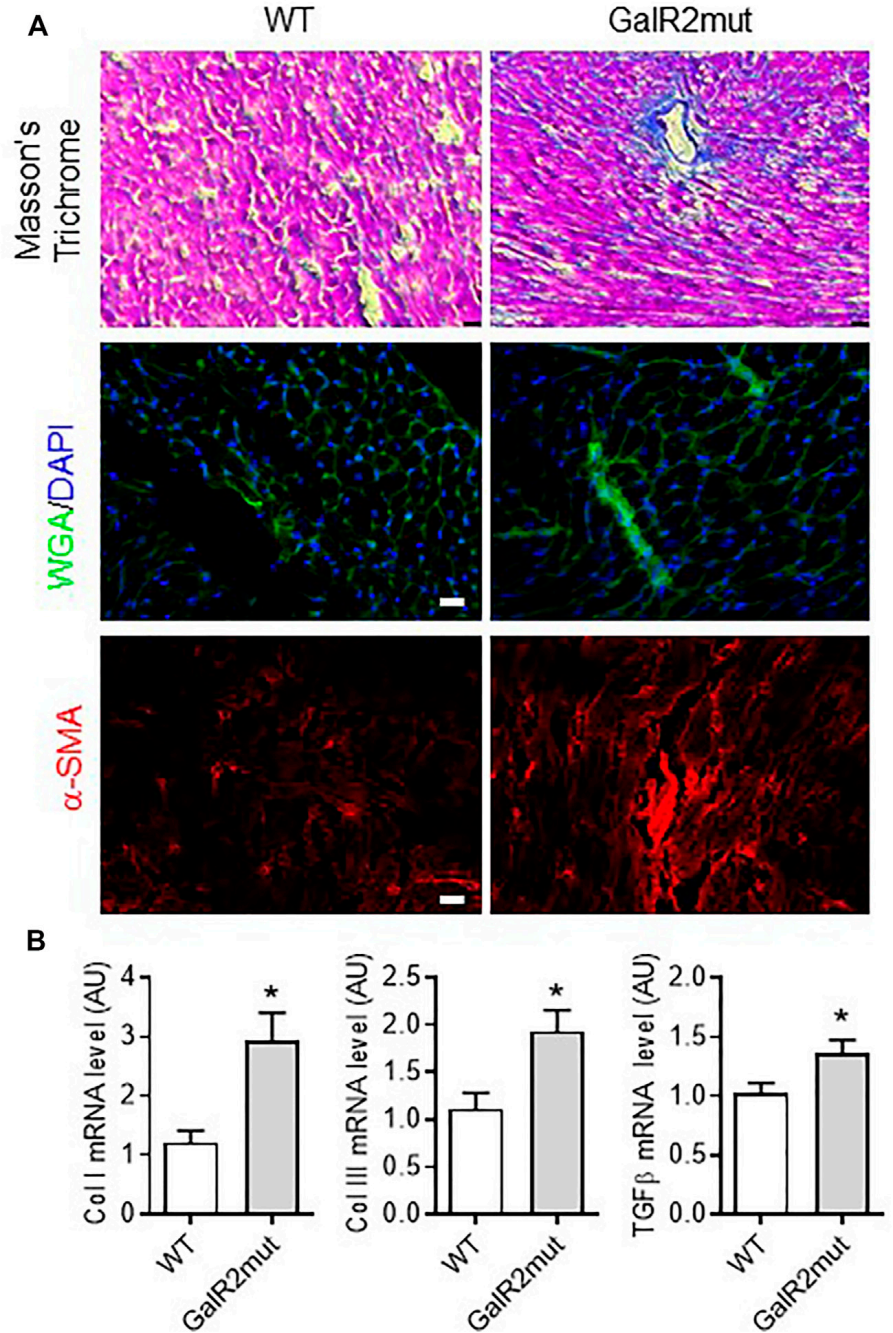
Cardiac fibrotic remodeling in GalR2 mutant mice. **(A)** Representative images of Masson’s Trichrome, WGA- or α-SMA immunolabelling or staining to depict fibrotic remodeling in GalR2mut hearts. **(B)** Myocardial mRNA levels of profibrotic factors Col I, Col III and TGFβ in WT and GalR2mut mice, *n* = 5–10. Data represents the mean ± SEM. *p < 0.05 vs. WT.

siRNA against GalR2 were from Sigma and as follows: 5′-GCT​CTG​GTC​TCC​AAG​CAT​T-3′ and 5′-GCU​CUG​GUC​UCC​AAG​CAU​U-3′. siRNA universal negative controls were from Sigma. Gene expression was assessed using quantitative polymerase chain reaction as described (Cinato et al., 2021). Briefly, total RNAs were isolated from rat embryonic cardiomyoblastic cell line H9C2 or mice heart using the RNeasy mini kit (Qiagen). Total RNAs (300 ng) were reverse transcribed using High Capacity cDNA Reverse Transcription Kit (Applied Biosystems) in the presence of random hexamers. The expression of target mRNA was normalized to HPRT (Figures 1, 3), β2-MG (Supplementary Figure S1) or RPLPO (Figures 2, 4) mRNA expression. Primers for qRT-PCR used in this study are as detailed in Table 1.

**TABLE 1:** Real-time qPCR primers.

Mouse GalR1	Forward 5′-GAA​ACT​AAG​GAA​AAC​AAG​AGC​CG-3′
	Reverse 5′-ACT​TGA​TAA​TTC​GCT​CCG​CC-3′
Mouse GalR2	Forward 5′-CAA​CCT​TGA​GTA​GAA​CCC​TCG-3′
	Reverse 5′-AAT​CCT​CGG​TCT​TTA​GCT​GC-3′
Mouse GalR3	Forward 5′-TTG​CCC​TCA​TCT​TCC​TGT​TG-3′
	Reverse 5′-AGG​ATG​AAG​CAA​AGG​TCG​G-3′
Rat GalR1	Forward 5′-TCG​GGA​CAG​CAA​CCA​AAC-3′
	Reverse 5′-TGC​AGA​TGA​TTG​AGA​ACC​TTG​G-3′
Rat GalR2	Forward 5′-CCT​GTT​CAT​CCT​CAA​CCT​GG-3′
	Reverse 5′-GCG​TGC​ATA​GTG​AGA​AAG​ATG-3′
Rat GalR3	Forward 5′-AGT​ACC​TAG​GAC​TGA​GGA​AGA​TG-3′
	Reverse 5′-AGT​AGC​ACA​GCC​AAC​ACC-3′
Mouse ANP	Forward 5′-AGA​GTG​GGC​AGA​GAC​AGC​AAA-3′
	Reverse 5′-AAG​GCC​AAG​ACG​AGG​AAG​AAG-3′
Mouse b-MHC	Forward 5′-AGG​TGG​CTC​CGA​GAA​AGG​AA-3′
	Reverse 5′-TGA​GCC​TTG​GAT​TCT​CAA​ACG​T-3′
Mouse BNP	Forward 5′-GCA​CAA​GAT​AGA​CCG​GAT​CG-3′
	Reverse 5′-CCC​AGG​CAG​AGT​CAG​AAA​C-3′
Mouse NRF1	Forward 5′-CAT​CTC​ACC​CTC​CAA​ACC​C-3′
	Reverse 5′-TGA​ATT​AAC​CTC​CTG​TGG​CG-3′
Mouse ATP6	Forward 5′-TCC​TAT​TCC​CAT​CCT​CAA​AAC​G-3′
	Reverse 5′-CAT​GTT​CGT​CCT​TTT​GGT​GTG-3′
Mouse COX1	Forward 5′-CCC​AGA​TAT​AGC​ATT​CCC​ACG-3′
	Reverse 5′-ACT​GTT​CAT​CCT​GTT​CCT​GC-3′
Mouse/Rat HPRT	Forward 5′-TGA​AAG​ACT​TGC​TCG​AGA​TGT​CAT-3′
	Reverse 5′-TCC​AGC​AGG​TCA​GCA​AAG​AA-3′
Mouse RPLPO	Forward 5′-TGA​CAT​CGT​CTT​TAA​ACC​CCG-3′
	Reverse 5′-TGT​CTG​CTC​CCA​CAA​TGA​AG-3′
Rat RPLPO	Forward 5′-GTC​ACA​GTA​CCT​GCT​CAG​AAC-3′
	Reverse 5′-CCA​CCT​TGT​CTC​CAG​TCT​TTA​TC-3′
Rat β2-MG	Forward 5′-CTG​GTC​TTT​CTA​CAT​CCT​GGC-3′
	Reverse 5′-ATA​GAG​CTT​GAT​TAC​ATG​TCT​CGG-3′

### Evaluation of Mitochondrial ROS Production

Mitochondrial O_2_^−^ production was measured using MitoSOX Red indicator (Life Technologies) as described (Tronchere et al., 2017). Briefly, cells were loaded with MitoSOX (2 µm) for 30min, washed in PBS, PFA-fixed, mounted on glass slide using MOWIOL and imaged on a Zeiss LSM780 confocal microscope. Frozen heart cryosections were hydrated in PBS and incubated with MitoSOX (5 µm) for 30 min at 37°C in a humidified chamber, quickly washed and imaged on a Zeiss LSM780 confocal microscope. The MitoSOX fluorescence intensity was quantified using Image J software on thresholded images.

### Statistical Analysis in *in Vivo* and *in vitro* Studies

Data are expressed as mean ± SEM. Comparison between two groups was performed by a two-tailed unpaired Mann-Whitney test while comparison of multiple groups was performed by Kruskal–Wallis one-way ANOVA followed by a Dunn’s post hoc test using GraphPad Prism version 9.3.1 (GraphPad Software, Inc.).

## Results

### GalR2 Expression Predominates in Cardiac Tissues and Cells

To gain insights into the role of the galaninergic system in cardiac cells, we first examined the expression of GalR1, GalR2 and GalR3 in tissues from adult mice and mouse cardiac myocytes. As shown in Figure 1, while the expression of the three galanin receptors was readily detected in spleen, only GalR2 mRNA could be detected in mice heart and primary cardiomyocytes. In addition, we found that GalR2 mRNA is present in H9C2 rat cardiomyoblasts (Supplementary Figure S1A).

### GalR2 Deficiency Promotes Cardiac Hypertrophic Remodeling in Mice

To further decipher the role of GalR2 in cardiac phenotype *in vivo*, we examined whether loss of GalR2 could affect hypertrophy, a hallmark of myocardial remodeling in mice. As shown in Figures 2A–C, mice deficient for GalR2 (GalR2mut) promoted cardiac hypertrophic phenotype as evidenced by cross sectional area in H&E and WGA-stained heart sections as compared with control WT mice. GalR2mut-dependent hypertrophy was confirmed by the expression of hypertrophic markers including β-MHC, ANP and BNP (Figures 2C–E).

### GalR2 Suppression Mediates Abnormal Mitochondrial Metabolism in Mice

Mitochondria are an important source of ROS in cardiac cells (Rosca et al., 2013). Therefore, we next examined myocardial mitochondrial ROS level in cardiac tissue from GalR2 deficient mice using MitoSOX Red, a fluorogenic dye highly selective for detection of superoxide production. As shown in Figure 3A, cardiac tissue from GalR2mut mice displayed elevated levels of mitochondrial ROS compared to WT mice heart. These findings suggest that GalR2 deficiency promotes cardiac hypertrophic remodeling and mitochondrial oxidative stress in the heart. In addition to mitochondrial ROS status, we measured myocardial expression of genes related to mitochondrial biogenesis. As shown in Figure 3B, we found that GalR2 deficiency in mice evokes a downregulation of genes linked to mitochondrial biogenesis including nuclear respiratory factor 1 (NRF1), ATP6 and COX1, suggesting that the GalR2 pathway regulates mitochondrial biogenesis in the heart.

### GalR2 Deficiency Favors Myocardial Fibrotic Remodeling in Mice

To further characterize the cardiac phenotype of GalR2 deficient mice, we analyzed the collagen deposition and localization on left ventricles from GalR2mut mice. As shown in Figure 4A, loss of GalR2 in mice promoted collagen accumulation in interstitium and perivascular regions within the left ventricle as evidenced by Masson’s trichrome and WGA-staining. Consistent with the reactive tissue fibrosis in mice deficient for GalR2, we demonstrated increased expression of α-smooth muscle actin (α-SMA), a biomarker for myofibroblast differentiation, in GalR2mut hearts compared to WT hearts (Figure 4A). Moreover, fibrosis signatures of heart tissue from GalR2mut mice was confirmed by the up-regulation of pro-fibrotic genes including Col I, Col III and TGFβ (Figure 4B). These findings suggest that GalR2 deficiency promotes cardiac fibrotic remodeling in the left ventricle.

### Galanin Orchestrates Cell Hypertrophic and ROS-Associated Cell Responses *via* GalR2

**FIGURE 5:**
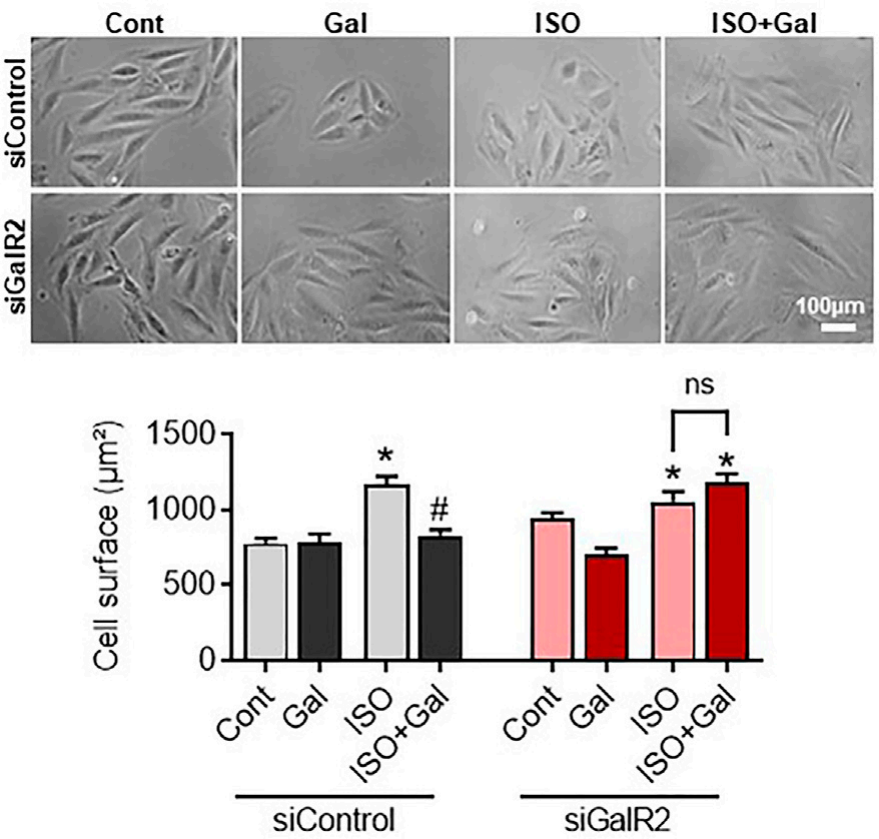
Galanin dictates cell hypertrophic responses *via* GalR2. Representative images and cell surface quantification of H9C2 cardiomyoblasts transfected with control siRNA (siControl) or GalR2 siRNA (siGalR2) under isoproterenol-induced stress (ISO). Data represents the mean ± SEM from three independent experiments. *p < 0.05 vs. control; #*p* < 0.05 vs. ISO.

**FIGURE 6:**
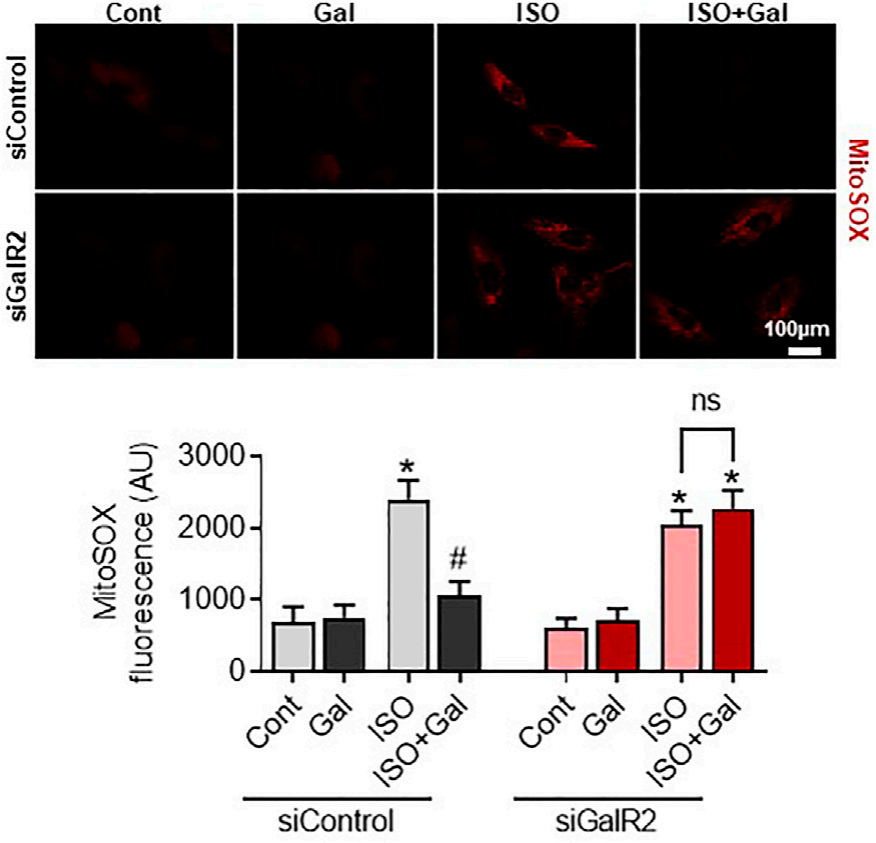
Galanin decreases mitochondrial ROS production *via* GalR2. Representative images and quantification of MitoSox Red staining in H9C2 cells transfected with control siRNA (siControl) or GalR2 siRNA (siGalR2) under isoproterenol-induced stress (ISO). Data represents the mean ± SEM from three independent experiments. *p < 0.05 vs. control; #*p* < 0.05 vs. ISO.

We have recently demonstrated that galanin inhibits mitochondrial ROS production and hypertrophy in cardiomyoblasts (Martinelli et al., 2021; Timotin et al., 2017). Therefore, we next examined whether the siRNA-mediated knockdown of GalR2 could affect galanin-mediated effects on cardiomyoblast hypertrophy and ROS accumulation induced by isoproterenol (ISO) treatment. In H9C2 cells, efficient GalR2 knocck-down was demonstrated by qPCR (Supplementary Figure S1B). As shown in Figure 5, galanin attenuated ISO-induced hypertrophy in control siRNA-transfected cells, whereas these effects were abolished in the GalR2 siRNA-transfected cells. Furthermore, galanin-induced effects on ROS generation following ISO treatment were suppressed in GalR2 siRNA transfected H9C2 cells (Figure 6). These data suggest that galanin drives hypertrophic responses and ROS production through GalR2.

## Discussion

Myocardial hypertrophic remodeling is the most common structural aberration linked to HF and sudden cardiac death (Konstam et al., 2011). In the hypertrophied hearts, mitochondrial ROS overproduction is associated with maladaptive ventricular reprogramming and decline in cardiac function. Despite the progress in understanding the pathophysiology of cardiac remodeling processes leading to HF, treatment options remain limited due to a lack of insight into the mechanisms orchestrating myocardial damage. Here, we report that galanin governs hypertrophic and ROS-associated responses to stress *via* GalR2. We provide the first evidence that genetic suppression of GalR2 promotes cardiac hypertrophy, fibrosis and mitochondrial oxidative stress *in vivo*. These findings provide new insights into the role of galaninergic system in cardiac remodeling and open news therapeutic avenues in heart protection.

Galanin, a multifaceted bioactive peptide, is widely distributed throughout the central and peripheral nervous system and some peripheral tissues (Crawley, 1995). Most of the biological functions of galanin remain unclear; however, galanin is mainly involved in modulating cognition, feeding, memory and neuroendocrine control (McDonald and Crawley, 1997; Díaz-Cabiale et al., 2005; Díaz-Cabiale et al., 2010; Lerner et al., 2010; Millón et al., 2017). We recently reported that galanin induces cardioprotective effects against myocardial I/R injury in animals (Pisarenko et al., 2017; Martinelli et al., 2021). Administration of this peptide prevented cardiomyocyte apoptosis and improved metabolic status of the myocardium *in vivo* (Timotin et al., 2017). The molecular actions of galanin are mediated through three galanin receptors subtypes, GalR1, GalR2 and GalR3 belonging to G protein coupled receptors, and signaling *via* multiple transduction pathways, including inhibition of cyclic AMP/protein kinase A (GalR1, GalR3) and stimulation of phospholipase C (GalR2) (Howard et al., 1997; Wang et al., 1997). In the present study, we found that GalR2 is the predominant receptor expressed in mouse cardiac cells and tissues. Comparative analysis of mRNA levels of galanin receptor family revealed that cultured myocytes and heart tissues express three galanin receptor subtypes with the prevailing pattern of GalR2 subtype. These data suggest that GalR2 could be involved in the function of galanin in cardiac cells. Previous study reported that GalR2 transcript is widely distributed in both central and peripheral tissues, whereas the expression of GalR1 is more restricted to brain and spinal cord (Kerr et al., 2015). The rat model showed the detectable levels of GalR2 mRNA in the heart demonstrating the significant role of GalR2 in the regulating cardiovascular responses (Kerr et al., 2015). Pharmacological or genetic manipulation of specific receptor subtypes and subsequent phenotypic changes should provide further insight into the relevant GalRs involved in cardiovascular control.

While the anatomical distribution and the distinct galanin receptor subtypes have been described, it remains unclear which galanin receptor or combination of galanin receptors may be mediating the cardiovascular effects of galanin. Previously, the experiments with knockout mice have explored the role of the GalR2 receptor in the anxiety-related behaviors, learning and memory, feeding, and analgesia (Bailey et al., 2007; Lu et al., 2008; Saar et al., 2011; Kerr et al., 2015). Despite the powerful assumption that would implicate galanin/GalR2 axis in the regulation of various complex behaviors, analysis of the GalR2 null mutants did not reveal a discernible phenotype. Gottsch et al. reported that GalR2 deficient mice exhibit normal growth, reproduction, body weight regulation, learning and memory, and susceptibility to seizure induction (Gottsch et al., 2005). It is possible that developmental mechanisms compensate for the congenital lack of GalR2 signaling or that redundant pathways mask the phenotype of the null mutation in GalR2. Alternatively, it is conceivable that GalR2 plays only a subtle role in these complex behaviors and that genetic deletion of GalR2 primes a phenotype that falls below detectable limits of the assays used to analyze these physiological processes. The present experiments elucidated the role of GalR2 in cardiac structural alterations and oxidative stress status in mice. Phenotypic analysis of mice deficient for GalR2 reveals a role for GalR2 in coordination of cardiac hypertrophy, fibrosis and mitochondrial ROS status. We found that suppression of GalR2 induces myocyte hypertrophy and abnormal expression of hypertrophic markers including ANP, BNP and β-MHC. Furthermore, we demonstrated that loss of GalR2 in mice leads to abnormal collagen accumulation within the myocardium and cardiac fibroblast differentiation into myofibroblasts reflecting fibrotic remodeling in GalR2mut mice. Importantly, we also shown that GalR2 deficiency in mice evokes a downregulation of NRF1, ATP6 and COX1, suggesting that GalR2 may have a role in the regulation of cardiac mitochondrial bioenergetic function. These data strongly suggest that GalR2 alone is sufficient to mediate perturbations in mitochondrial metabolic status and cardiac remodeling processes including hypertrophy and fibrosis. Further studies will be required to determine the exact functions of GalR2 in cardiovascular homeostasis, and whether it is indeed part of a pathway that could coordinate myocardial mitochondrial function.

Recently, we demonstrated that galanin plays a critical role in cardiac cell survival in the failing heart (Martinelli et al., 2021), however, the receptor subtypes that mediate the protective effects of galanin in cardiac cells are unclear. Here we report that galanin protects against hypertrophy and mitochondrial oxidative stress through GalR2-dependent pathway. Using siRNA knockdown in cardiomyoblasts, we show that galanin can reverse the effects of galanin on isoproterenol-induced hypertrophy and mitochondrial ROS generation. These findings imply that galanin dictates anti-hypertrophic and anti-oxidant effects through GalR2 pathway. Our findings are consistent with previous studies demonstrating that GalR2 is the predominant receptor subtype that transduces the neuroprotective effects of galanin (Elliott-Hunt et al., 2007). Respectively, GalR2-specific agonist would have utility in various forms of brain or heart damage, either reducing or minimizing cell death.

In summary, these findings unravel new insights into the role of galanin/GalR2 axis in the heart and suggest novel therapeutic strategies in heart disease. We report that GalR2 is the predominant receptor subtype in the myocardium orchestrating the antihypertrophic and ROS-suppressing actions of galanin.

## Data Availability

The original contributions presented in the study are included in the article/Supplementary Materials, further inquiries can be directed to the corresponding author.
