# Supplementary figures and images for: Galanin Regulates Myocardial Mitochondrial ROS Homeostasis and Hypertrophic Remodeling Through GalR2
Source: Front Pharmacol. 2022 Mar 31;13:869179. doi: 10.3389/fphar.2022.869179 (PMC9011366; doi:10.3389/fphar.2022.869179)

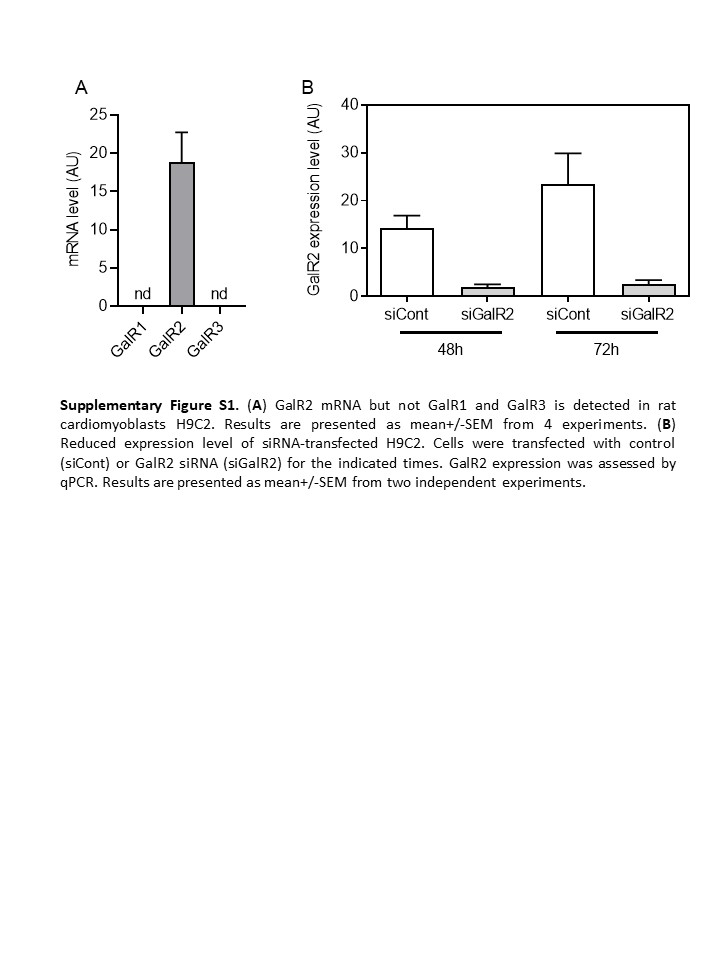

Supplement: Supplementary file 1 [file Image1.JPEG]
